# Supplementary material for: Private Keeping of Dangerous Wild Animals in Great Britain
Source: Animals (Basel). 2024 May 7;14(10):1393. doi: 10.3390/ani14101393 (PMC11117220; doi:10.3390/ani14101393)
Supplement: Supplementary file 1 [file animals-14-01393-s001.zip › animals-2963376-supplementary.pdf]

Supplementary Materials

Table S1. overview of taxa licensed to be kept under DWAA in 2020.

**Birds**				
Family	Genus	Species kept under licence	Count of licences	Population
Casuariidae	*Casuarius*	1	1	4
Struthionidae	*Struthio*	1	18	330
**Invertebrates**				
Araneae				
Family	Genus	Species kept under licence	Count of licences	Population
Ctenidae	*Phoneutria*	-	2	72
Sicariidae	*Loxosceles*	≥2	4	73
Theridiidae	*Latrodectus*	-	2	72
Scorpiones				
Family	Genus	Species kept under licence	Count of licences	Population
Buthidae	*Androctonus*	≥4	3	6
	Centruroides	1	1	1
	Leiurus	1	3	6
	Orthochirus	1	1	2
	Parabuthus	2	2	2
	Rhopalurus	1	1	1
	Unknown	-	2	300
Unknown	Unknown	-	1	14
**Mammals**				
Artiodactyla				
Family	Genus	Species kept under licence	Count of licences	Population
Bovidae	*Antilope*	1	4	95
	Bison	≥2	9	282
	Connochaetes	≥1	1	10
	Damaliscus	1	1	10
	Kobus	2	3	37
	Madoqua	≥1	1	2
	Oryx	≥1	1	10
	Taurotragus	≥1	1	10
	Tragelaphus	2	2	28
Camelidae	*Camelus*	2	12	37
Cervidae	*Rangifer*	1	2	6

Hippopotamidae	*Hexaprotodon*	1	2	4
Suidae	*Sus*	1	14	1034
Carnivora				
Family	Genus	Species kept under licence	Count of licences	Population
Ailuridae	*Ailurus*	1	2	3
Canidae	*Canis*	1	6	30
	Chrysocyon	1	1	2
	Speothos	1	1	2
Eupleridae	*Cryptoprocta*	1	1	5
Felidae	*Acinonyx*	1	3	10
	Caracal	1	4	10
	Catopuma	1	2	3
	Felis	2	3	5
	Herpailurus	1	2	6
	Hybrid	N/A	27	103
	Leopardus	1	4	6
	Leptailurus	1	34	75
	Lynx	≥2	10	26
	Neofelis	1	2	7
	Panthera	5	4	30
	Pardofelis	1	1	2
	Prionailurus	3	6	16
	Puma	1	5	18
	Uncia	1	2	3
Hyaenidae	*Crocuta*	1	1	2
	Hyaena	1	1	3
Mustelidae	*Amblonyx*	1	4	7
	Eira	1	1	2
	Gulo	1	1	2
	Martes	1	1	6
	Mellivora	1	1	2
	Melogale	1	1	6
	Unknown	1	1	4
Ursidae	*Helarctos*	1	1	2
Diprotodontia				
Family	Genus	Species kept under licence	Count of licences	Population
Macropodidae	*Macropus*	1	1	7
Perissodactyla				

Family	Genus	Species kept under licence	Count of licences	Population
Equidae	*Equus*	≥3	10	39
Tapiridae	*Tapirus*	≥1	5	12
Primates				
Family	Genus	Species kept under licence	Count of licences	Population
Atelidae	*Ateles*	1	1	5
	Unknown	≥1	2	3
Cebidae	*Cebus*	2	2	5
	Sapajus	1	6	10
	Unknown	≥1	10	26
Cercopithecidae	*Macaca*	≥3	3	27
	Pygathrix	≥1	1	3
	Cercopithecus	1	1	1
	Chlorocebus	2	2	9
	Colobus	1	1	9
	Papio	1	1	2
Hylobatidae	*Nomascus*	≥1	1	2
Lemuridae	*Eulemur*	6	3	15
	Hybrid	1	1	1
	Lemur	1	23	101
	Varecia	≥2	10	34
Pitheciidae	*Chiropotes*	1	1	10
	Unknown	≥1	1	4
Unknown	Unknown	≥1	1	6
Proboscidea				
Family	Genus	Species kept under licence	Count of licences	Population
Elephantidae	*Elephas*	1	1	2
**Reptiles**				
Crocodilia				
Family	Genus	Species kept under licence	Count of licences	Population
Alligatoridae	*Alligator*	2	11	54
	Caiman	2	10	22
	Paleosuchus	1	12	19
Crocodylidae	*Crocodylus*	4	3	55
	Osteolaemus	1	4	6
Gavialidae	*Tomistoma*	1	1	2
Squamata - Venomous Lizards				

Family	Genus	Species kept under licence	Count of licences	Population
Helodermatidae	*Heloderma*	≥3	16	106
Squamata - Venomous Snakes				
Family	Genus	Species kept under licence	Count of licences	Population
Colubridae	*Rhabdophis*	1	1	1
Elapidae	*Acanthophis*	1	2	2
	Aspidelaps	2	9	14
	Dendroaspis	≥4	4	7
	Elapsoidea	1	1	1
	Hemachatus	1	1	1
	Naja	16	22	103
	Ophiophagus	1	9	14
	Oxyuranus	≥1	2	2
Lamprophiidae	*Malpolon*	2	3	7
Viperidae	*Agkistrodon*	4	13	26
	Atheris	1	6	9
	Atropoides	2	3	8
	Bitis	3	7	14
	Bothriechis	1	5	8
	Bothrops	≥3	6	10
	Calloselasma	1	1	1
	Cerastes	1	4	9
	Crotalus	≥17	26	144
	Daboia	3	4	9
	Deinagkistrodon	1	3	5
	Echis	2	8	14
	Gloydius	1	1	1
	Macrovipera	1	1	2
	Montivipera	2	3	4
	Protobothrops	1	1	2
	Sistrurus	2	7	15
	Trimeresurus	9	13	33
	Tropidolaemus	1	3	4
	Vipera	3	10	23
Unknown	Unknown	-	3	15

Table S2: changes in the total number of DWAA licensed animals since 2000. Farmed species = bison, boar and ostrich; plus, emu, vicugna and guanaco pre-2007. Other species = all other species on the DWAA Schedule.

Study	Total Licensed Dangerous Wild Animals	Farmed Species	Other Species
2001<br>(Greenwood et al.)	11878	10555	1323<br>(11%)

England & Wales		(89% of Total DWAA)	
2010<br>(Copping – Sunday Telegraph)<br>England only	4296	3130<br>(73%)	1166<br>(27%)
2017<br>(Born Free Foundation)<br>England & Wales	4331	2776<br>(64%)	1555<br>(36%)
This study<br>England & Wales	3695	1590<br>(43%)	2105<br>(57%)

Table S3. percentage change of the most commonly kept taxa in England and Wales under the DWAA since 2000 (Scotland omitted for comparison with Greenwood et al. [3]*). Comparisons for certain taxa such as primates is not possible due to the number of species removed from the Schedule in 2007 and how numbers were reported in the 2001 Greenwood et al. study.*

Category	Taxa	2000 DWAA Population	2020 DWAA Population	% Change
Other Species	Crocodilians	50	149	+198%
	Scorpions	11	232	+2009%
	Venomous snakes	258	500	+94%
	Wild cats	169	266	+57%
Farmed Species	Ostrich	4769	330	-93%
	Wild boar	4554	1034	-77%

Table S4. *species identified as being a "Greater Risk" of causing serious injury or a threat to the life of the public under the SSSMZP, but do not currently require a DWAA licence to house privately. Excludes obligate aquatic species (e.g., cetaceans, fish, molluscs) where public risk from escape into a terrestrial environment is unlikely. Species are approximate reflecting discrepancies in taxonomy* [1,4].

**Family**	**Taxon**	**Common name**	**Number of species in taxon (approx.)**
Bradypodidae	*Bradypus*	Three-toed sloths	4
Megalonychidae	*Choloepus*	Two-toed sloths	2
Phyllostomidae	Desmodontinae	Vampire bats	3
Cervidae	*Cervus*	Red deer, wapiti, sika deer (and adult males of other species)	8
	Elaphurus	Pere David's deer	1
	Capreolus	Roe deer (adult male)	2
Cathartidae	*Vultur*	Andean condor	1
	Gymnogyps	Californian condor	1
Accipitridae	*Gyps*	Vultures and Griffon vultures	8
	Aegypius	European black vulture	1
	Torgos	Lappet-faced vultures	1
	Harpia	Harpy eagle	1
	Harpyopsis	New Guinea harpy eagle	1
	Pithecophaga	Monkey-eating eagle	1
	Stephanoaetus	Crowned eagle	1
	Polemaetus	Martial eagle	1
Strigidae	*Bubo*	Eagle owls (adults breeding or with young)	18
Bucerotidae	*Bucorvus*	Ground hornbills	2
Chelydridae	*Chelydra*	Snapping turtle	3
	Macroclemys	Alligator snapping turtle	1-2
Varanidae	*Varanus komodoensis*	Komodo dragon	1
Boidae (and *Pythonidae*)		Pythons and boas (all specimens over 3m)	8-11
